# Strong Discrepancies between Local Temperature Mapping and Interpolated Climatic Grids in Tropical Mountainous Agricultural Landscapes

**DOI:** 10.1371/journal.pone.0105541

**Published:** 2014-08-20

**Authors:** Emile Faye, Mario Herrera, Lucio Bellomo, Jean-François Silvain, Olivier Dangles

**Affiliations:** 1 Institut de Recherche pour le Développement (IRD), UR 072, Laboratoire Evolution, Génomes et Spéciation, UPR 9034, Centre National de la Recherche Scientifique (CNRS), Gif sur Yvette, France et Université Paris-Sud 11, Orsay, France; 2 UPMC Univ Paris06, Sorbonne Universités, Paris, France; 3 Facultad de Ciencias Exactas y Naturales, Pontificia Universidad Católica del Ecuador, Quito, Ecuador; 4 Mediterranean Institute of Oceanography (MIO) CNRS/INSU, IRD, UM 110, Université de Toulon, La Garde, France; 5 Instituto de Ecología, Universidad Mayor San Andrés, Cotacota, La Paz, Bolivia; Clemson University, United States of America

## Abstract

Bridging the gap between the predictions of coarse-scale climate models and the fine-scale climatic reality of species is a key issue of climate change biology research. While it is now well known that most organisms do not experience the climatic conditions recorded at weather stations, there is little information on the discrepancies between microclimates and global interpolated temperatures used in species distribution models, and their consequences for organisms’ performance. To address this issue, we examined the fine-scale spatiotemporal heterogeneity in air, crop canopy and soil temperatures of agricultural landscapes in the Ecuadorian Andes and compared them to predictions of global interpolated climatic grids. Temperature time-series were measured in air, canopy and soil for 108 localities at three altitudes and analysed using Fourier transform. Discrepancies between local temperatures vs. global interpolated grids and their implications for pest performance were then mapped and analysed using GIS statistical toolbox. Our results showed that global interpolated predictions over-estimate by 77.5±10% and under-estimate by 82.1±12% local minimum and maximum air temperatures recorded in the studied grid. Additional modifications of local air temperatures were due to the thermal buffering of plant canopies (from −2.7°K during daytime to 1.3°K during night-time) and soils (from −4.9°K during daytime to 6.7°K during night-time) with a significant effect of crop phenology on the buffer effect. This discrepancies between interpolated and local temperatures strongly affected predictions of the performance of an ectothermic crop pest as interpolated temperatures predicted pest growth rates 2.3–4.3 times lower than those predicted by local temperatures. This study provides quantitative information on the limitation of coarse-scale climate data to capture the reality of the climatic environment experienced by living organisms. In highly heterogeneous region such as tropical mountains, caution should therefore be taken when using global models to infer local-scale biological processes.

## Introduction

Bridging the gap between the predictions of coarse-scale climate models and the fine-scale climatic reality of species is increasingly recognized as a key issue of climate change biology research [1], [2], [3], [4]. Despite decades of study on microclimates [5], [6], [7], [8] and evidence for habitat-related and topographical variations in local temperatures and their relevance for species ecology [2], [9], [10], [11], [12], [13], most attempts to understand and model species distributions still do not integrate spatially-explicit fine-scale climatic data (e.g. [14], [15], [16]). Many work use global model of temperature interpolation to examine species vulnerability to climate change and, doing so, ignore the critical issue of habitat complexity in climate buffering [4], [5], [17]. Indeed, climate surfaces used in species distribution models (SDMs) are rarely generated or interpolated to a resolution finer than 1 km^2^ (e.g. WorldClim database), a resolution that is still very coarse relative to the home ranges or body size of most species [13], [18]. For instance, [8] showed that climate grid lengths used in SDMs are, on average, ∼10,000-fold larger than studied animals, and ∼1,000-fold larger than studied plants. Their meta-analysis showed that the WorldClim was the most widely used climatic dataset in global SDMs. As this commonly used coarse scale climatic data in SDMs overlook the spatiotemporal thermal heterogeneity experienced by organisms, there is an urgent need for a more sophisticated use of these datasets for making inferences about biological processes that are driven by hour to hour operative temperatures of organisms.

An important yet poorly studied issue in climate change biology is to quantify to what extent climatic conditions differ between widely used 1 km^2^ interpolated grid cells of global climatic database and real-world landscapes of similar areas. While it is now well-known that most organisms, especially tiny ectotherms such as insects and other arthropods, do not experience the climatic conditions recorded at weather stations [9], [12], [18], there is little quantitative information on the spatial and temporal heterogeneity at the landscape scale of local climatic conditions (i.e. conditions at biologically relevant scales, e.g., from cm to km for insects) and their consequences for organisms’ performance. A better quantification of the climatic conditions of ecologically-relevant habitats over relatively large landscape scales (e.g., 1 km^2^) is therefore a necessary first step to better incorporate dynamical microclimate into global distribution models.

Here, we investigate the sources of variance between global interpolated and local temperatures by examining 1) how well WorldClim predicts local air temperatures in our study region (the tropical Andes), 2) to what extent temperatures in crop canopies and soils differ from local air temperatures, and 3) how relevant is to use WorldClim to infer the potential performance of an insect crop pest. Addressing these questions is not an easy task as the mosaic of climatic habitats relevant for small ectothermic species at a 1-km^2^ scale in real-world landscapes may be outstandingly complex. In this study, we focused on highland agricultural landscapes of the tropical Andes as most prior similar data came from low elevation and temperate agroecosystems. In such systems, most crop pests experience, over their entire life cycle, climatic conditions in three well-defined environmental layers (air, air inside-canopy and soil) and these conditions are remarkably stable over the year [19]. In this context, we firstly decided to map over replicated 1-km^2^ climatic grid cells the ecologically relevant local temperatures for ectothermic crop pests in agricultural landscapes, and to compare these maps to interpolated temperature grid cells of the widely used WorldClim database. We used Fourier analysis applied to local temperature time-series as a tool to fit daily variations of temperature and to feature microclimate discrepancies in space and in time (both in terms of amplitude and phase). We then explored the implication of our thermal landscape mapping for pest performance by comparing temperature frequencies in our grid cells with the temperature-dependent growth curve of the potato tuber moth (*Phthorimaea operculella*) a major crop pest species in the region and worldwide.

## Materials and Methods

### 1. Study area

The Ecuadorian Andes are characterized by a low seasonality, with mean temperatures varying more within days (up to 30°K variation) than within months and years (less than 0.6°K and 0.2°K variations, respectively, see [19]). This region exhibits a marked altitudinal gradient in temperatures (between 2000 and 4000 m) with mean monthly air temperature roughly decreasing by 0.6°K every 100 m of elevation [20]. Agricultural landscapes dominate the altitudinal belt between 2600 and 3800 m, and are typically composed by small field crops (mainly potato *Solanum tuberosum* L., broad bean *Vicia faba* L., corn *Zea mays* L., alfalfa *Medicago sativa* L., and pasture), natural grasslands (páramos) and a few forest patches [21]. Under the climatic conditions of the region, crops can be planted and harvested all year round, thereby creating a landscape mosaic of a wide variety of crops at different phenological stages.

Our study area was located 115 km south from the equatorial line (01°01′36″S, 78°32′16″W) in the Cotopaxi province of Ecuador. It spread out on a 20-km^2^ elevation transect (2.35×8.5 km), ranging from 2,600 to 3,800 m a.s.l. The gradient had a Southwest exposure and an average slope of 9.5° (±5.2) (based on a 30 m resolution digital elevation model). To investigate the elevation effect on local vs. global interpolated temperature variations, we divided our study area into three 400 m altitudinal belts which correspond to natural floors in the hillside (2,600–3,000 m, 3,000–3,400 m, and 3,400–3,800 m) with a mean monthly temperature of 13.2±0.4°C, 10.8±0.6°C, and 9.3±0.4°C, respectively. Beyond temperature, these belts also differed in terms of landscape composition (Appendix S1 in Supporting Information), with lower elevations dominated by small fields (0.3±0.1 Ha) of potato, corn, broad bean, and pasture while the higher band had larger fields (0.7±0.3 Ha) of mainly potato and pasture. Working in these agricultural landscapes no requires specific permissions expect the kind agreement of the field owner. The presented study did not involve endangered or protected species.

### 2. Temperature data collection

In each of the three-altitudinal belts, we measured temperature regimes in six habitats (five crops and natural grasslands) where insect pests can be found. In each habitat, we defined three layers: air, air inside-canopy (referred as “air canopy” in the text) and soil. These layers are all used by most insect pests over their life cycle: air layer by adults, air canopy layer by adults and leaf-eating larvae and pupae, soil layer by tuber feeding larvae and pupae. In each layer of each habitat, temperature was recorded with a 1 min time step using data loggers (Hobo U23-001-Pro-V2 internal temperature loggers, Onset Computer Corporation, Bourne, USA) with an accuracy of ±0.21°K over the 0–50°C range and a resolution of 0.02°K at 25°C. According to [4], 1) air loggers were fixed on a wooden stake at 1 m high to overstep most crop canopies and sheltered by a 20 cm^2^ white plastic roof to minimize solar radiation heating; the roof was itself placed 5 cm above the logger to avoid warming by greenhouse effect, 2) air canopy loggers were placed 0.3 m high inside vegetation 5 cm bellow large leaves to minimize the effect of direct solar radiation and 3) soil loggers were buried 0.1 m into the ground where roots and tubers grow (see Appendix S2 for photographs). In each field, only one logger per layer measured the temperatures. Those triplets of loggers were located at the centre of the field to avoid edge effect (see Appendix S3 for an analysis of the spatial variability of temperatures within a field and [22]). As vegetation land cover influences microclimate beneath and around plants, see [5], [6], we repeated these 54 measurements (3 elevations ×6 habitats ×3 layers) for three classes of leaf area index (LAI) [23] defined as follows: 0 (bare soil), 0.01–0.5 for and >0.5 of LAI. Minimum LAI was fixed to 0.01 to avoid confusion with bare soil and allowed enough leaf area to place the loggers underneath. At each measurement site, LAI values were visually estimated (twice) measuring the ratio of leaf area within a 1-m^2^ quadrant sub-divided into 0.1 m^2^ cells delimited by strings. This indirect method did not account for leaves that lie on each other however it relates to shaded areas that influence inside-canopy and soil microclimates [23].

Each of the 162 measurement combinations (3 altitudinal belts ×6 habitats ×3 layers × 3 LAI classes) was replicated 1–3 times depending on availability of habitats at a given elevation and phenology stage. In total 324 independent temperature time series were acquired over 15 days between September and December 2011 (data available in Appendices S9, S10 and S11). Importantly, under the climatic conditions of the study area, 15-days time series characteristics did not differ from those obtained over one year (see Appendix S4 for details). At each measurement site, we recorded the UTM-WGS84 geographic coordinates with a handheld GPS Garmin Oregon 550 (Garmin, Olathe, USA).

### 3. Global solar radiations

Infrared and visible radiations (expressed in Watt/m^2^) were monitored in each altitudinal belts using a LI-1400 LI-COR datalogger equipped with a LI-200 pyranometer sensor (LI-COR, Lincoln, USA) placed perpendicular to gravity. Between 9∶00 AM and 4∶00 PM, mean global solar radiations ranged from 500 to 1000 watts/m^2^, with temporal variability mainly induced by short-term changes in cloud cover.

### 4. Data analyses

#### 4.1. Times series analyses using Fourier transforms

**Figure 1:**
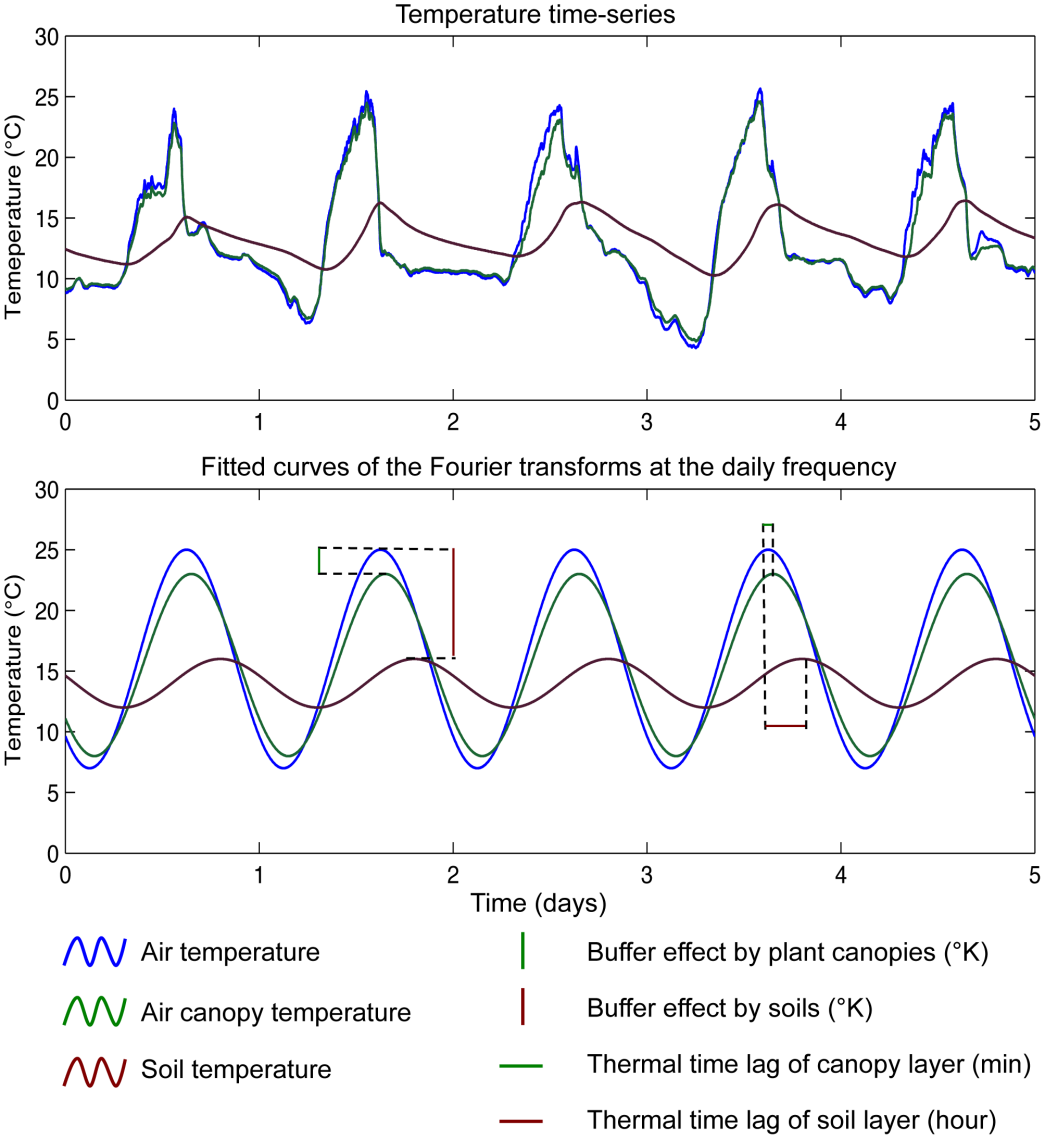
Fit of temperature time series with discrete Fourier transforms at the daily frequency *K_d_*. Air temperatures are in blue, crop canopy temperatures are in green and soil temperatures are in brown.

Air and air canopy temperature time series showed extreme events during a few minutes that were certainly due to strong radiations experienced at the study sites − these affected loggers recording despite their plastic roofs. Therefore, we found relevant to fit our time series data with a discrete Fourier transform (DFT) at the daily frequency *k_d_* (Fig. 1) as this allowed averaging daily minimum and maximum temperatures while limiting the effect of short extremes (mainly for maximum). Moreover fitting temperature time series with the DFT allowed us to circumvent (or partially resolve) the issue of comparing time series with different temporal resolution: a sinusoid built from a daily time step time series will be accurate enough to compare with another sinusoid built from a one minute time step time series (our operative temperatures vs. global climatic models).

DFT analyses allowed us estimating two important descriptors of the time series at the daily frequency *k_d_*: the amplitude *A_d_* and the phase φ*_d_* of the DFT (see Appendix S5 for details). The thermal amplitude allowed us to measure the thermal buffer effect in Kelvin between air and canopy layers and air and soil layers (Fig. 1 and Appendix S5). The phase allowed us to measure the thermal time lag expressed in minute in inside-canopy and soil layers with respect to the air layer (Fig. 1 and Appendix S5). Thermal time lag therefore quantifies the time delay in time series to reach their maximum between air vs. canopy and air vs. soil layers. This is an important climatic parameter to test whether microclimate conditions below canopy (canopy and soil layers) would track air conditions with some time lag depending on habitat characteristics.

We also ran DFT analyses on a four-year monitoring (2008–2012) of air temperatures (recorded at one meter high with half an hour time step with the same shelter process described above) to measure the seasonality. Analyses were performed for the three-altitudinal belts of the study area (2800, 3200, 3600 m) by reading the amplitude at the seasonal frequencies (91, 182 and 364 days, see Appendix S6). On average the Fourier transform amplitudes at 91, 182 and 364 days were 0.14 (+/−0.01), 0.44 (+/−0.04), 0.97 (+/−0.03)°K indicating that the seasonality was negligible in the study area [24].

All Fourier analyses were performed in MATLAB R2011a (Mathworks, Natick, USA). The effects of habitat, elevation, LAI classes and the interaction “elevation × LAI classes” on daytime and nigh-time DFT amplitudes and on DFT thermal time lag were assessed using a two-way ANOVA with Bonferroni corrections. When habitat was found significant, we ran post-hoc multiple comparisons using a Tukey HSD test to identify differences among habitats. All statistical analyses were performed in R version 3.0.0 (R Development Core Team 2012).

#### 4.2. Thermal landscape analyses

To compare local temperatures with global interpolated climate data employed in species distribution models, we considered one of the most widely used and readily available climate database, WorldClim [25]. The WorldClim database is a set of global climate layers (interpolated averages of monthly minimum, maximum and mean 1.5 m high air temperatures from weather stations spread out worldwide) with a spatial resolution of 30 arc seconds. Close to equator, this resolution is equivalent to squares of 0.86 km. In each altitudinal belt, we selected one WorldClim grid cell with homogenous slope (between 5.4° and 7.9°), micro-topography and exposition (south-west). Based on a digitized municipal cadastre (from the town council of Salcedo, Cotopaxi province) and a 5-m resolution digital orthophoto (Ecuadorian Military Geographical Institute, www.igm.gob.ec/site/index.php), we built the digital landscape of each grid cell in ArcGIS 10.01 (ESRI, Redlands, USA). In addition to the six studied habitats, crop storage infrastructures were also included into the digital maps as they significantly modify air temperature patterns, offering optimal conditions for crop pest development [26]. Outside air vs. inside air storage-temperature relationships for different elevations were derived from measurements made by [26] within the same area with similar temperature data design (see Fig. 1 in Appendix A2 of their paper). Roads and woodlots were also indicated on the maps even if they were not included in the temperature comparison analysis, as they do not constitute relevant habitats for crop pests.

In order to simulate landscape thermal heterogeneity, crop habitats were attributed with one crop type (potato, broad bean, corn, alfalfa or pasture) and one LAI classes (0, 0.01–0.5, >0.5) based on a survey of 85 sites in the region, in which we quantified landscape composition (% of each crop and LAI classes) in 100-m radius sampling circles (see Appendix S7). For each habitat, we assigned the corresponding air, air canopy and soil temperature values at each elevation. Finally, since we were particularly interested in minimal and maximal values, as they are the most biologically relevant for ectothermic crop pests [4], we focused on minimum and maximum temperatures obtained from the DFT analyses and the WorldClim database.

Afterwards, we decomposed the variance of temperatures between global interpolated grids and local temperatures measured in agricultural landscapes by mapping the differences in minimum and maximum temperatures between the air local temperatures (Air *_L_*) and the WorldClim interpolated temperatures (Air *_WC_*) for the three studied grid cells. Then, to illustrate the part of the variance due to microclimate effects, we mapped the differences in minimum and maximum temperatures between measured local air canopies, soil temperatures (Layer _L_) and the air local temperatures (Air *_L_*) for the three studied grid cells.

#### 4.3. Pest performance in thermal landscape

As a final step of our analysis, we explored the implication of our thermal landscape mapping for pest performance by comparing temperature frequencies in our grid cells with the temperature-dependent growth curve of a major crop pest species in the region: *Phthorimaea operculella* (Lepidoptera: Gelechiidae). This pest is considered one of the most important potato pests worldwide, but also attacks a wide variety of other crops such as tomato (*Solanum lycopersicum* L.), eggplant (*Solanum melongena* L.) or tobacco (*Nicotiana tabacum* L.) (see [27] for a review). *P. operculella* feeds on different part of the plant (leaves, stems, and tubers) and also tubers in storage structures [26], [28]. In agricultural landscapes, *P. operculella* is abundant in virtually all types of habitats (even far from its host plant) because 1) this pest is able to fly over large distances (100–250 m) to infest suitable host plants [29] and 2) a significant quantity of tubers are left in the field after harvest, and are rapidly colonized by the moth before the following crop is planted. It is therefore common to observe infested potato plants in corn or broad bean fields. These left-over potatoes are well know by farmers and agronomists as significant obstacle to the control of these pests [28].

The temperature-dependent growth rate curve of *P. operculella* larvae (in day-1) over a 0–40°C range was obtained using published temperature-response data of laboratory experiments performed in the Andean region (see [30] for details). PTM development rate data were then modeled with the [31] equation as modified by [32]:
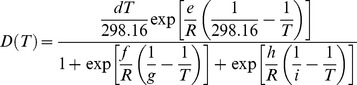
(1)where *T* is temperature in Kelvin (°C+273.15), *R* = 1.987, and *d*, *e*, *f*, *g*, *h*, and *i* estimated parameters. This model has been widely used to describe the kinetics of insect development based on several assumptions about the underlying developmental control enzymes. For instance, it has been used to describe poikilotherms’ temperature-dependent development [33].

We then compared the growth rate performance curve of *P. operculella* for local temperature distribution (canopy and soil layer temperatures) and for global interpolated ones (e.g., Fig. 3 in [3]). Distributions of canopy and soil minimum, maximum and mean temperatures were extracted from the three digitized landscapes using the geostatistical analyst extension of ArcGIS. Canopy and soil temperature frequencies were expressed as the percent of total grid cell area. The growth performance model of *P. operculella* given by Eqn. 1 was implemented with WorldClim minimum and maximum temperatures and the local minimum, maximum and mean temperature distribution. This allowed estimating insect growth rate within the range of WorldClim and measured field data.

## Results

### 1. Local vs. global air temperature discrepancies in thermal landscapes

**Figure 2:**
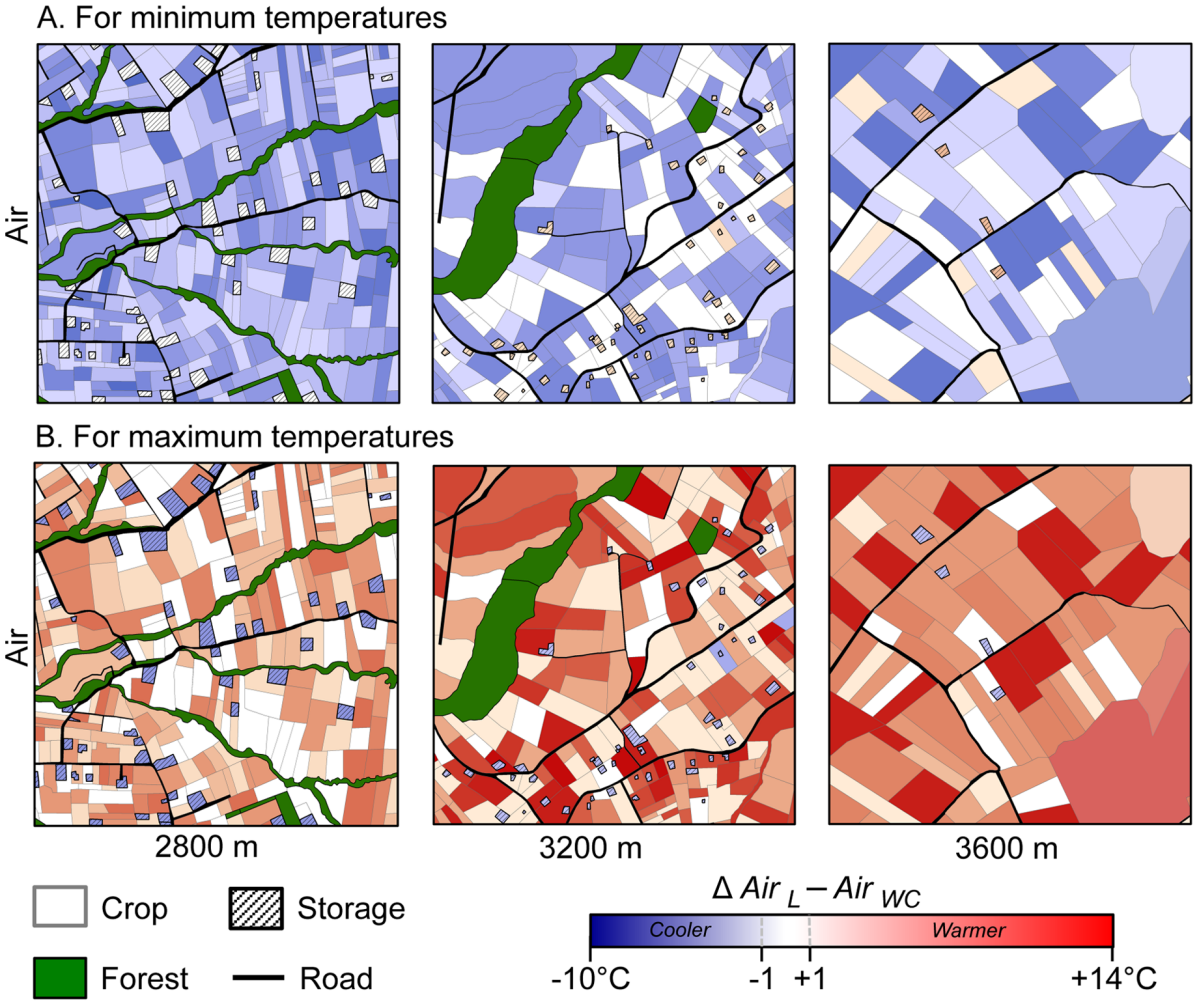
Maps showing the differences between local air temperatures and the WorldClim interpolated minimum (A) and maximum (B) *(Δ Air _L_ − Air _WC_*). Blue colours indicate *Δ Air _L_* ***−*** *Air _WC_* <0, i.e. area where local air temperatures are cooler than those gave by WorldClim. Red colours indicate *Δ Air _L_* ***−*** *Air _WC_* >0, i.e. area where air local temperatures are warmer than the ones gave by the WorldClim. White colours *Δ Air _L_* ***−*** *Air _WC_* = 0 indicate areas where air WorldClim temperatures equate air local temperatures (±1°C). The extent and position of each square is equal to the spatial resolution of the WorldClim database: 30-arc sec that is the equivalent of 0.86 km^2^ for the study area. Temperatures in storages were obtained from [26].

Differences in average minimum and maximum temperatures between local air temperatures and the global coarse grain interpolated air temperatures from the WorldClim (*Δ* Air *_L_* – Air *_WC_*) were mapped for the three studied grid cells (Fig. 2). While minimum local air temperatures were cooler than those predicted by WorldClim in 77.5±10% of the studied areas (blue areas, average min Δ Air *_L_* – Air *_WC_* = −2.9°K) maximum local air temperatures were warmer than extrapolated temperatures in 82.1±12% of the studied areas (red areas, average max Δ Air *_L_* – Air *_WC_* = +5.6°K). This pattern was not influenced by elevation. Notably, for all elevations, local mean air temperatures were quite well predicted by the WorldClim (+/−1°K) as in average 55.3±3.4% of the studied areas felt in the range of Air *_L_* – Air *_WC_* ≤1°K (Appendix S8).

### 2. Temperature discrepancies due to microclimate in agricultural landscapes

**Figure 3:**
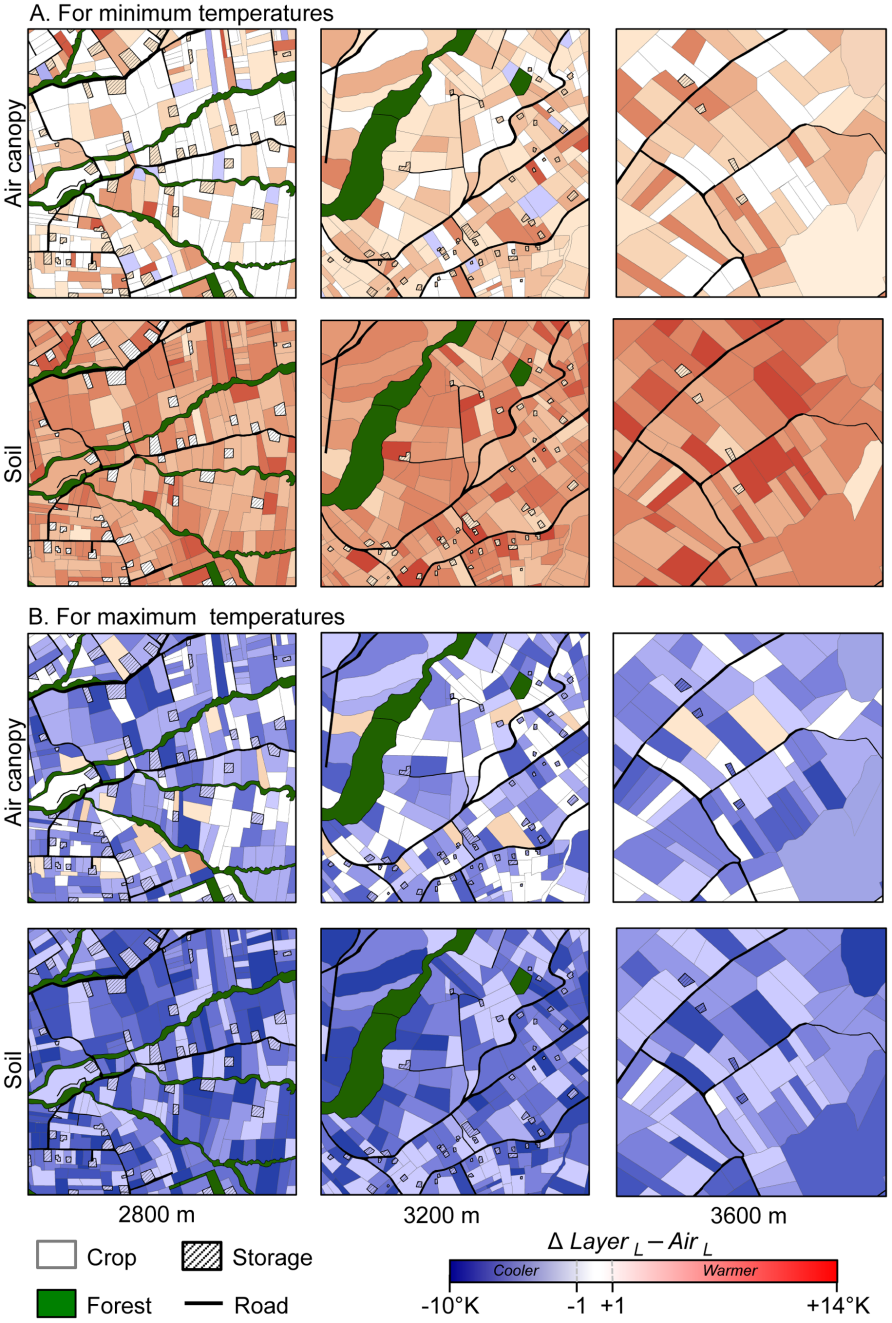
Maps showing the differences between local air canopy and soil temperatures with the air local for minimum (A) and maximum (B) *(Δ Layer _L_ − Air _L_*). Colour code is given in Figure 2.

**Figure 4:**
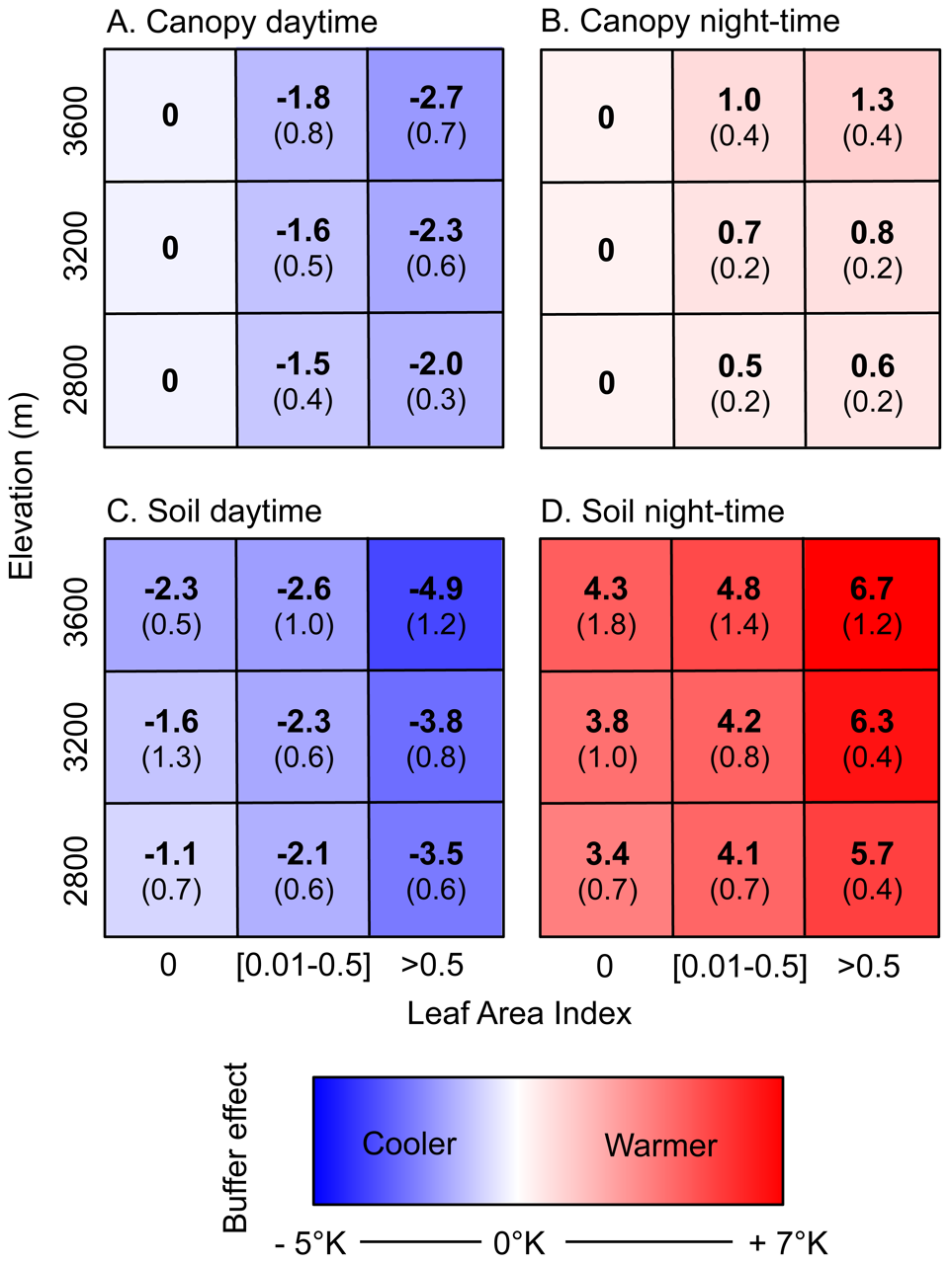
Mean thermal buffering from Fourier transforms at the daily frequency for canopy (A, B) and soil temperatures (C, D) as a function of elevation and leaf area index. (A, C) show the daytime temperature excursion with respect to air, whereas (B, D) are the equivalent results for night-time temperatures. The 95% interval of confidence is given between brackets. Blue colours show colder temperatures than air. Red colours show warmer temperatures than air.

Differences in average minimum and maximum temperatures between local canopy and soil temperatures and local air temperatures (*Δ* Layer _L_− Air *_L_*) were mapped for the three studied grid cells (Fig. 3). Overall, canopy and soil areas were always cooler than maximum air temperature and were always warmer than air minimum temperatures resulting in a general buffer effect of minimum and maximum air temperatures by canopy and soil layers. The buffer effect on air temperatures was significantly stronger for soil than for canopy layer (see Fig. 4, Student’s t-test, *t* = −27.10 and *t* = 4.52, *P<0.001* for night-time and daytime, respectively). Interestingly, the buffer effect on air temperatures by soil was higher during night-time than daytime (Fig. 4D) while the opposite pattern was found in crop canopy (Fig. 4A).

Elevation had a significant effect on air temperature buffering in the canopy layer but not in the soil layer (Table 1). Contrastingly, LAI had a highly significant thermal buffering effect in both soils (night and daytime) and canopies (daytime, see Table 1). Buffer effect on air temperatures by bare soil (e.g. without plant cover, LAI = 0) ranged from −1.1°K to −2.3°K for daytime and from 3.4°K to 4.3°K for night-time. Crop type had no significant effect on buffering patterns except for potato in which higher buffer effects were recorded (Post-Hoc HSD test, P<0.05).

**Table 1:** Results of the two-way ANOVA with a Bonferroni correction on the effects of habitat, elevation, LAI and elevation × LAI terms on daytime and nigh-time DFT amplitudes and thermal time lag on inside-canopy and soil temperature time series.

Effect	Canopy				Soil			
	Df	Mean sq	F value	P value	Df	Mean sq	F value	P value
Daytime amplitude								
Habitat	5	6.282	3.370	0.007*	5	5.745	2.466	0.036
Elevation	2	12.491	6.701	0.002*	2	5.722	2.456	0.089
LAI	1	40.171	21.551	<0.001*	1	136.78	58.705	<0.001*
Elevation × LAI	2	2.513	1.348	0.263	2	0.292	0.125	0.882
Residuals	132	1.864			127	2.330		
Night-time amplitude								
Habitat	5	0.936	3.895	0.002*	5	1.390	0.839	0.525
Elevation	2	4.539	18.896	<0.001*	2	2.143	1.293	0.278
LAI	1	1.083	4.509	0.035	1	157.52	95.041	<0.001*
Elevation × LAI	2	1.754	7.302	0.010	2	0.097	0.059	0.943
Residuals	132	0.240			127	1.657		
Thermal Time Lag								
Habitat	5	0.001	1.297	0.269	5	0.009	3.881	0.003*
Elevation	2	0.001	5.777	0.004*	2	0.024	10.139	<0.001*
LAI	1	0.001	29.322	<0.001*	1	0.022	9.165	0.003*
Elevation × LAI	2	0.001	2.374	0.097	2	0.005	2.334	0.101
Residuals	132	0.001			127	0.002		

Bold* indicates significant results (P<0.05).

**Figure 5:**
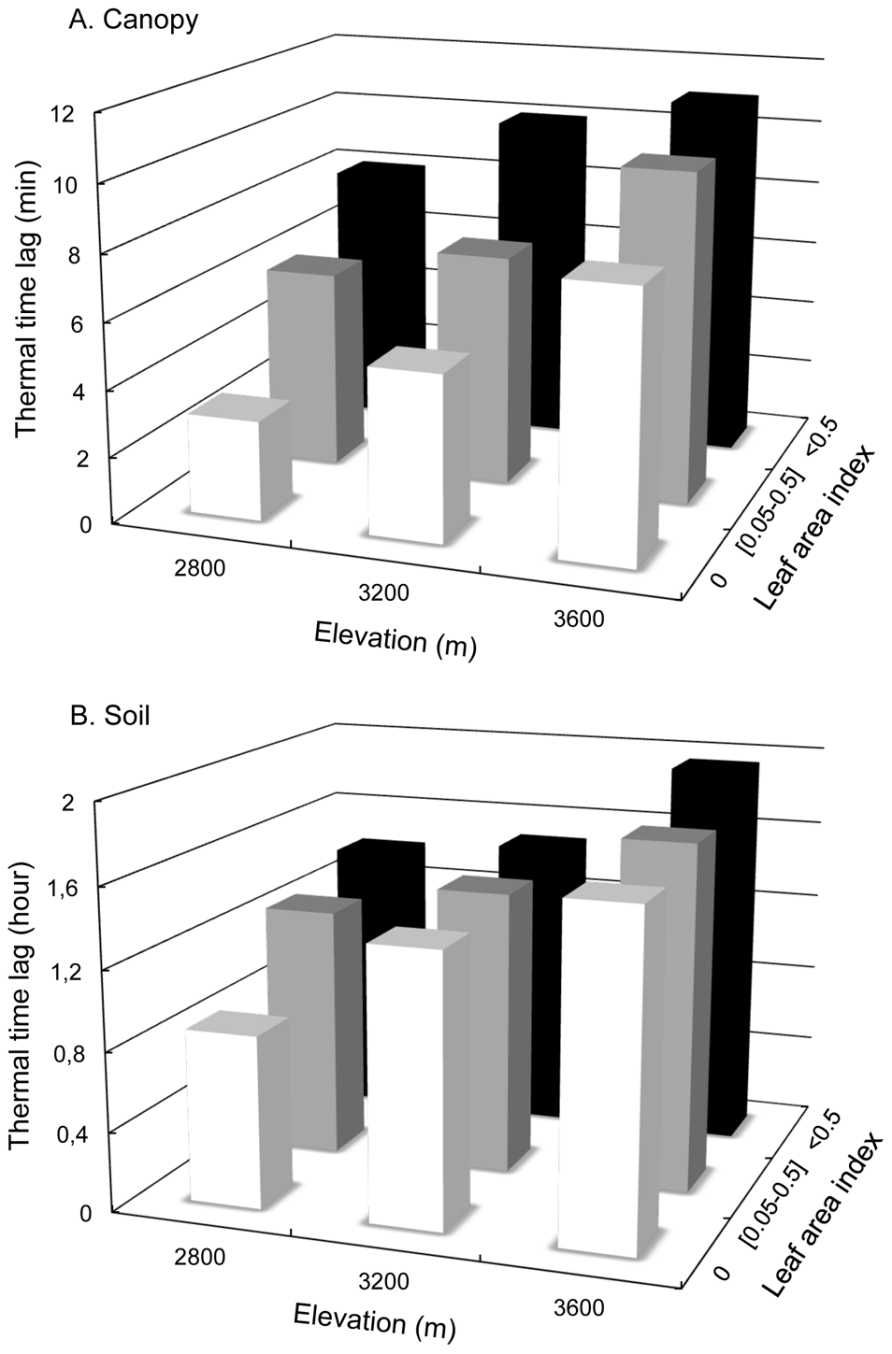
Thermal time lag from Fourier transforms at the daily frequency for canopy (A) and soil temperatures (B) as a function of elevation and leaf area index. The z-axis (log+1 transformed) is expressed in minutes (A) and in hours (B).

Overall, thermal time lag was much shorter in canopies (7.5±2.6 min) than in soils (1.5±0.3 hours, Fig. 5). LAI classes had a significant positive effect on thermal time lag for both canopy and soil layers (Table 1). On average, thermal time lag increased by 2 min. in canopies and 30 min. in soils between two LAI classes. Similarly, elevation had a significant positive effect on thermal time lag for both canopy and soil layers (Table 1) with an average increase of 2±0.3 min. in canopies and of 60±31 min. in soil between two altitudinal belts (Fig. 5).

### 3. Thermal performance curve using local vs. interpolated temperatures

**Figure 6:**
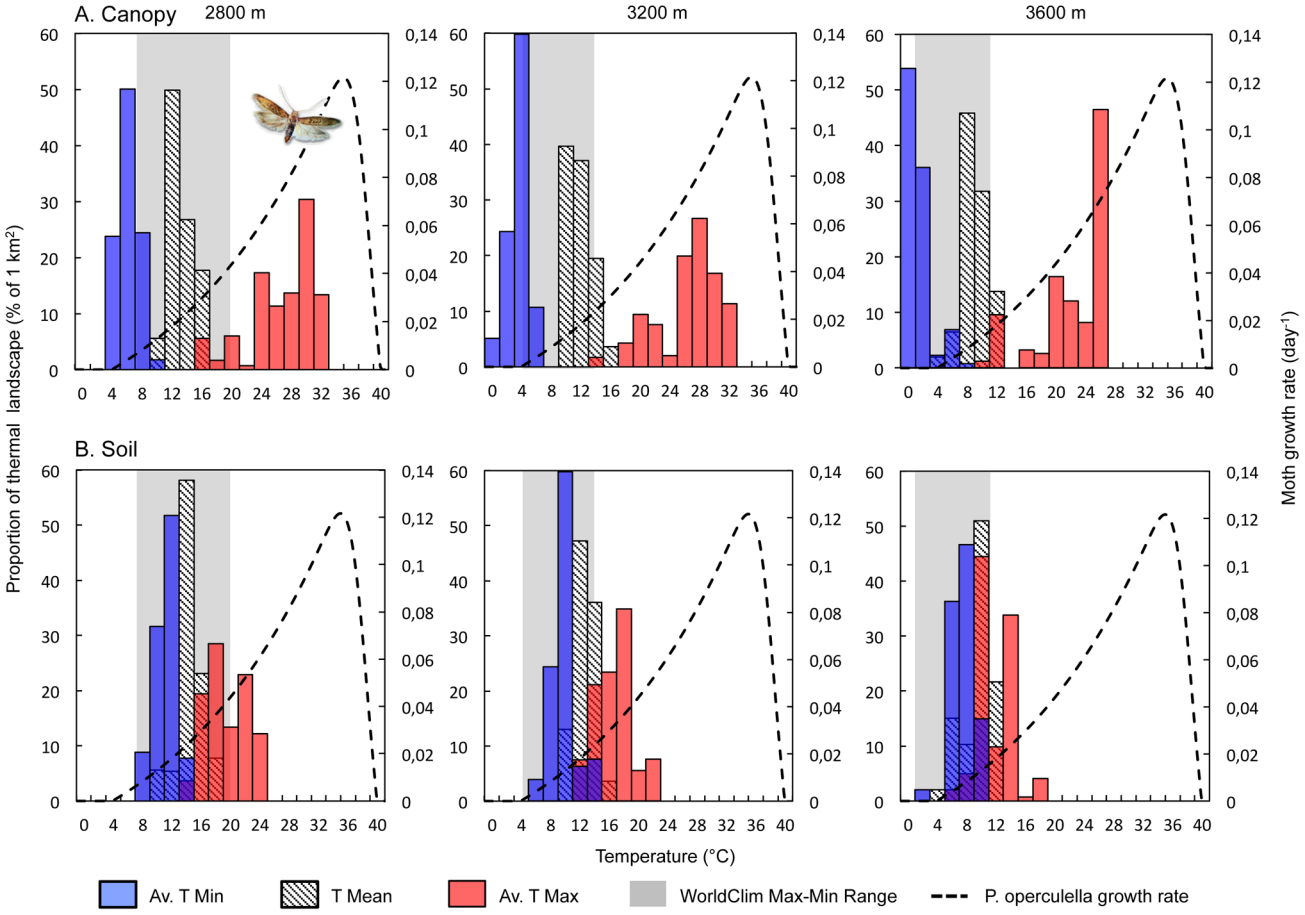
Superimposed plot of the temperature-dependent growth rate curve of the potato moth Phthorimaea operculella (dashed line) and the frequency distribution (% of area) of average minimum (blue), maximum (red) and mean (striped) temperatures for canopy and soil layers at the three studied elevations. Grey (shaded) bands in the background represent the WorldClim minimum and maximum temperature range.

To assess the implication of local vs. global interpolated temperature discrepancies for crop pest performances, we plotted the frequency distribution of the minimum (blue bars), maximum (red bars) and mean local (stripped bars) temperatures and those given by WorldClim (from minimum to maximum temperature, shaded region in the background) with the temperature-dependent growth rate curve of the potato moth *P. operculella* (Fig. 6). As a general pattern, global interpolated temperature ranges predicted lower growth rates of *P. operculella* than those predicted by local temperatures at all elevations, in both inside-canopy and soil layers (where the pest lives most of their time). While mean temperature distribution generally fell within the WorldClim min-max range, extreme temperatures (and especially maximum ones) largely exceeded this range.

The WorldClim estimations predicted *P. operculella* growth rates ranging between 0.007 and 0.045 day^−1^ at 2800 m, and between 0 and 0.018 day^−1^ at 3600 m, the maximum rates being slightly lower than those predicted by soil temperatures (0.068 day^−1^ at 2800 m and 0.037 day^−1^ at 3600 m). These differences were exacerbated in canopy layers where estimated maximum growth rates were 2.6–4.3 times higher than those predicted by WorldClim (0.118 day^−1^ at 2800 m and 0.079 day^−1^ at 3600 m). Discrepancies between WorldClim and local temperature-based growth rate estimations were not significantly affected by elevation (One-way ANOVA, *F* = 7.79, *P = 0.219* and *F* = 1.67, *P = 0.419* for canopies and soils, respectively).

## Discussion

Accurate predictions of the responses of organisms to climate change using SDMs require knowledge of microclimates at spatial and temporal scales relevant for studied organisms [13], [34],[35]. To our knowledge, our study is the first to quantify the thermal heterogeneity among a set of agricultural habitats at fine spatial and temporal scales and to compare those thermal microhabitats to the most widely used global climatic dataset in SDMs. By documenting the mosaic of thermal habitats found in tropical agricultural landscapes, our study confirms previous evidence that microclimates strongly differ from nearby macroclimates due to the variability of air motion and solar radiation patterns created by complex topographies with heterogeneous elevation, slope angle, exposure or roughness [1], [7], [18], [36]. Our study therefore supports the view that results from the long tradition of agrometeorological studies on microclimates (e.g. [6], [17], [22]) have to be revived in the new context of microhabitat modelling for predicting the response of organisms to climate change.

### 1. LAI-based and elevation-based climate heterogeneity

In contrast to many previous studies (see [7] for a review), our objective was not to examine the well-documented effect of topography on local temperatures but rather to examine the less-known effects of habitat types and vegetation land cover on thermal landscape features. We found significant thermal time lag and buffer effects on air temperatures by plant and soil layers below crop canopies during night-time and daytime. The top of canopies reflects and absorbs part of the solar radiation during the day, allowing less energy to reach the layers (plants and soils) below canopies. During the night, infrared heat released from both the ground and plants is partly held back by the canopy above [5]. As a consequence plants and soils limit night-time cooling and daytime warming [6], leading to a significant buffer effect of minimum and maximum temperatures [1], [4], [17]. That is also why we found a buffer effect on air temperatures by soil higher during night-time than daytime and the opposite pattern for crop canopies.

Our results indicate a strong effect of elevation on thermal buffering and thermal time lag by canopy and soil layers. This could result from the combination of a negative relationship between elevation and air temperature and a positive relationship between elevation and solar radiation exposure, part of which is absorbed by plants and soils [6]. As a result, the difference between air temperature and canopy and soil temperature increased with elevation. Interestingly, the modifications of local temperatures by habitats and LAI were of the same magnitude (from −2.70 to 4.82°C in average) than that generated by topography-related factors [7], [36], supporting the need to better consider habitat effects on microclimates.

### 2. Fine scale variations in temperature vs. climatic units

Our findings show that the complex agricultural mosaic resulting from habitat types and LAI classes at the landscape scale was a major modifier of the thermal patterns in the studied tropical highlands. More importantly, our findings revealed that, at best, 55% of landscape habitats had real mean air temperatures that were well estimated by WorldClim predictions while in average less than 20% of these areas had minimum and maximum air temperatures well estimated. Additional thermal discrepancies between large and fine-scale temperatures resulted from heterogeneity in crop types and phenologies. This strongly supports the view that the common use of the WorldClim database arrayed into 1-km^2^ grids may not adequately capture the reality of the climatic environment experienced by living organisms, in particular tiny ectothermic species [2], [3], [13], [18]. It is important to note that to obtain the highest level of thermal heterogeneity we chose a complex mountainous agricultural study area that provided boundary conditions for climate modelling. Indeed, these mountainous areas provide strong climatic gradients and extreme habitat fragmentation which combined with un-seasonal agrosystem make up a mosaic of thermal patches that expanded the difficulties to faithfully assess climatic parameters for modelling [25]. In view of the urgent need of fine scale climate data with large extent [2], [8], [35] more research is necessary to develop accurate up- or down-scaling methods, in mountainous locations where thermal heterogeneity is large, and may be needed to properly describe the ecologically significant microclimates [7], [37].

### 3. Microclimates and species distribution models

From tiny insects to mega-herbivores, it is well recognized that species ecology is strongly influenced by micro-climatic features of the landscape [2], [10], [11], [12], [13] yet quantitative information on how thermal landscape heterogeneity may affect species performance is scarce. Short-scale differences in temperatures may provide opportunities for individual organisms, even with limited dispersal capabilities, to escape unfavourable microclimates or to maximize physiological performances by selecting preferred microclimates [38], [39]. Our analysis showed that predictions on *P. operculella* growth rates strongly differed between Wordclim-based and locally-measured temperatures, suggesting that global species distribution models using global coarse-scale climatic datasets without further microclimate modelling could be strongly limited to accurately predict species occurrence and performance, in particular that of ectotherms living in habitats such as mountain slopes. Such a spatial heterogeneity in thermal patches, where climatic conditions are strongly modified, provides a mosaic of favourable, sub-optimal or lethal thermal habitats that directly influences the performance of natural populations of ectotherms.

Coarse-extent modeling of microclimate is currently one of the major obstacles to predicting how organism will react to their experienced environments and forecast their distribution under climate change [8]. To date, two main types of models have been shown to provide relatively accurate, continent-wide calculations of microclimate: statistical model and mechanistic model [13]. The first one is statistical as the variables are not deterministically but stochastically related. These models perform statistical correlation of species occurrences with climatic data and have proven to be powerful interpolative tools for defining and projecting climatic envelopes [40], [41]. A disadvantage of these statistical models is that they can only be applied to the conditions under which they are fitted. On the other hand, mechanistic models of the climatic responses of organisms [13], [34] use fundamental knowledge of the interactions between process variables to define the model structure. Therefore they do not require much data for model development and validation. One of them is the Microclim model recently developed by [35], [42] for all terrestrial landmasses − except Antarctica− which quantify key microclimatic parameters at macro-scales, with a relatively fine spatial (15 km^2^) and temporal resolution (hours). The microclimatic parameters such as wind velocity, humidity, and solar radiation allow building energy and mass budgets of organisms, and therefore serve as key inputs for biophysical models of species distributions.

It is important to highlight that a better spatiotemporal resolution in temperature patterns should go in pair with the development of more accurate temperature-based population dynamics models to integrate it [2], [13], [34], [43]. Existing predictions of models based on insect response measured in constant temperatures may yield different and less realistic results than those from predictions of models that include the effect of real temperature fluctuation on insect biology [33]. For example, to date, we still do not know the impact of a few hours of warm temperature for the performance of ectotherm species at longer time scales [33]. In this context, fine-scale spatiotemporal temperature mapping has revealed a key step for any studies aiming at understanding, predicting and managing the responses of species distributions to climate change.

## Supporting Information

Appendix S1 **Habitat and field size distribution in the three studied altitudinal belts.**
(PDF)

Appendix S2 **Photos of the temperature recording experiment.**
(PDF)

Appendix S3 **Spatial variability of temperatures within a field.**
(PDF)

Appendix S4 **Comparison of time series analysis outputs using 15 days vs. 1-year temperature data.**
(PDF)

Appendix S5 **Fourier analysis description.**
(PDF)

Appendix S6 **Seanonality measured on four year air temperature time series with Discrete Fourier Transform.**
(PDF)

Appendix S7 **Crop habitat composition survey used in the study area.**
(PDF)

Appendix S8 **Local and global air mean temperature discrepancies mapping.**
(PDF)

Appendix S9 **Microclimate temperature time-series used in this work #1.**
(ZIP)

Appendix S10 **Microclimate temperature time-series used in this work #2.**
(ZIP)

Appendix S11 **Microclimate temperature time-series used in this work #3.**
(ZIP)
