# Supplementary material for: Strong Discrepancies between Local Temperature Mapping and Interpolated Climatic Grids in Tropical Mountainous Agricultural Landscapes
Source: PLoS One. 2014 Aug 20;9(8):e105541. doi: 10.1371/journal.pone.0105541 (PMC4139370; doi:10.1371/journal.pone.0105541)
Supplement: Appendix S1 — Habitat and field size distribution in the three studied altitudinal belts. (PDF) [file pone.0105541.s001.pdf]

Appendix S1: Habitat and field size distribution in the three studied altitudinal belts.

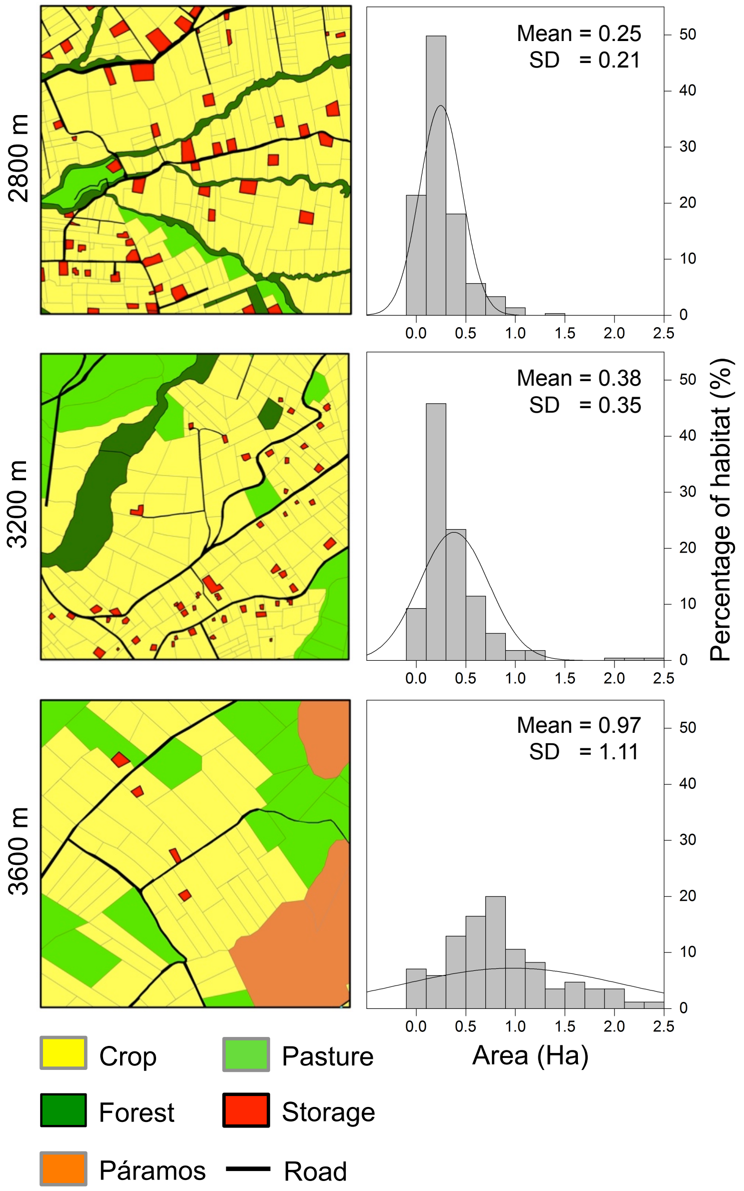

Figure S1: Habitat mapping of the three studied 1-km^2 grid cells at their respective elevations (2800, 3200 and 3600 m) and the corresponding frequency distribution histograms of field areas.
