# Supplementary material for: Strong Discrepancies between Local Temperature Mapping and Interpolated Climatic Grids in Tropical Mountainous Agricultural Landscapes
Source: PLoS One. 2014 Aug 20;9(8):e105541. doi: 10.1371/journal.pone.0105541 (PMC4139370; doi:10.1371/journal.pone.0105541)
Supplement: Appendix S2 — Photos of the temperature recording experiment. (PDF) [file pone.0105541.s002.pdf]

Appendix S2: Photos of the temperature recording experiment.

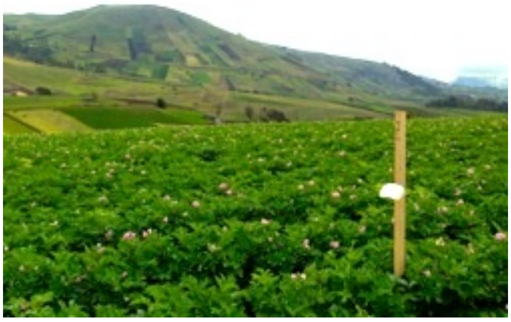

A.

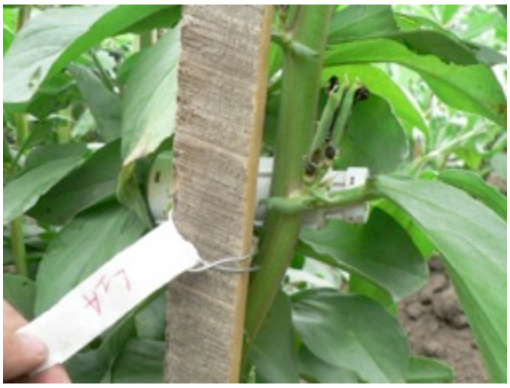

B.

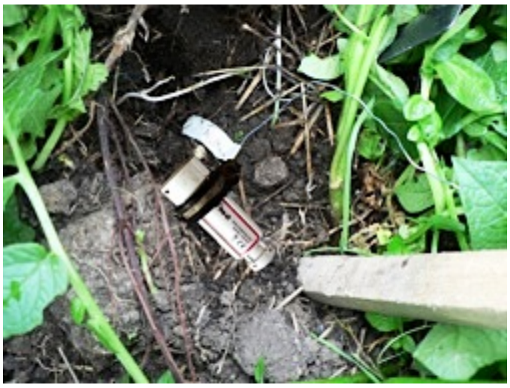

C.

Figure S2: Photos of the temperature recording experiment. Photograph A. 20 cm^2 white plastic shelter placed 5 cm above the air logger fixed on a wooden stake at 1 m high in a fully-grown potato field. Photograph B. Air canopy logger (Hobo U23-001 Pro V2 internal temperature loggers, Onset Computer Corporation, Bourne, USA) placed 0.3 m high inside vegetation 5 cm bellow large leaves in a fully-grown broad bean field. Photograph C. Soil logger (Hobo U23-001 Pro V2 internal temperature loggers, Onset Computer Corporation, Bourne, USA) placed 10 cm inside ground before burial.
