# Supplementary material for: Strong Discrepancies between Local Temperature Mapping and Interpolated Climatic Grids in Tropical Mountainous Agricultural Landscapes
Source: PLoS One. 2014 Aug 20;9(8):e105541. doi: 10.1371/journal.pone.0105541 (PMC4139370; doi:10.1371/journal.pone.0105541)
Supplement: Appendix S3 — Spatial variability of temperatures within a field. (PDF) [file pone.0105541.s003.pdf]

Appendix S3: Spatial variability of temperatures within a field.

Edge effect, micro-topography and LAI variations within a field can strongly change the microclimate of plant and soil layers creating heterogeneous thermal conditions at the field scale [6, 17, 22]. To address this issue, in a parallel experiment we measured air, air canopy and soil temperatures at six different locations within the same field. Measurements were replicated in 4 fields with area ranging from 596 to 672 m^2 of in order to capture to variability of field size in the study area. Fields were located between 2900 and 3000 m and were composed of fully-grown potatoes. Temperatures were recorded over one month using loggers (Hobo U23-001 Pro V2 internal temperature loggers, Onset Computer Corporation, Bourne, USA, 1 min time step) arranged as described in the main document (see part 2.2). Figure S3 shows that the discrete Fourier transformed amplitudes at the daily frequency of the one-month temperature time series did not vary among field location for both canopy and soil layers in the 4 replicate fields.

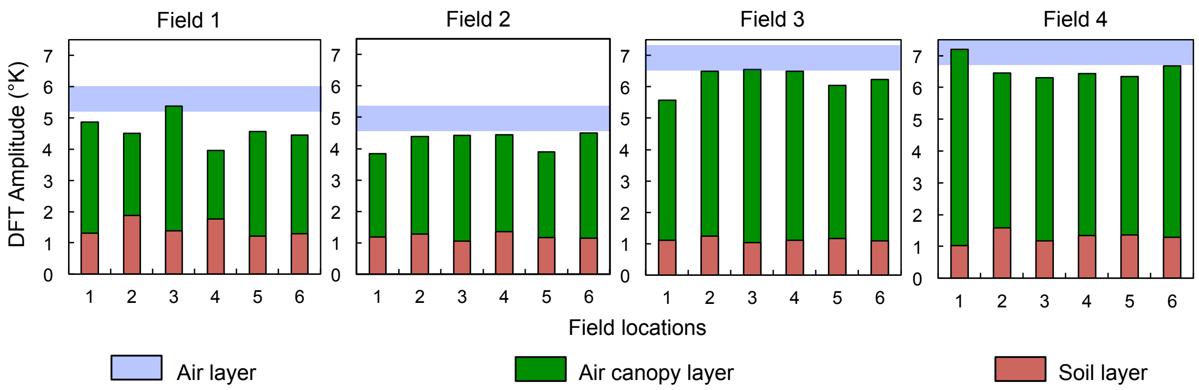

Figure S3: Histogram of the DFT amplitudes of air (light blue), canopy (green) and soil (brown) layers in the 4 fields studied.
