# Supplementary material for: Strong Discrepancies between Local Temperature Mapping and Interpolated Climatic Grids in Tropical Mountainous Agricultural Landscapes
Source: PLoS One. 2014 Aug 20;9(8):e105541. doi: 10.1371/journal.pone.0105541 (PMC4139370; doi:10.1371/journal.pone.0105541)
Supplement: Appendix S4 — Comparison of time series analysis outputs using 15 days vs. 1-year temperature data. (PDF) [file pone.0105541.s004.pdf]

Appendix S4: Comparison of time series DFT analyses outputs using 15 days vs. 1-year temperature data.

We assessed the relevance of using 15-days temperature time series as a good proxy of climatic conditions occurring over longer time scales (one year) using data from a four-year monitoring (2008-2012) of air temperatures, at three elevations in the study area. Air temperatures were measured using loggers (Hobo U23-001 Pro V2 internal temperature loggers, Onset Computer Corporation, Bourne, USA), covered by a plastic roof and fixed on a wooden stake 1 m high (see main document part 2.2 for details). Using the same Fourier transform analysis described in the main document, we then compared daily discrete Fourier transform amplitude A_d of 15-days air temperature time series vs. 1-year air temperature time series chosen randomly over the 4-year database. We ran between 10 and 50 pairs of time series (15 days vs. a year) for each elevation, the starting for each time series being chosen randomly among the three first years of the four-year air temperature data. We found a highly significant positive relationship between the amplitude of the 15-days and the 1-year Fourier transform at the daily period (see Fig S4). The slope of the 15-days vs. 1-year curve did not significantly differ from the 1:1 slope (ANCOVA, df =114, $F = 2.08$, $p > 0.05$). The small variations observed between both slopes are likely the result of sporadic meteorological phenomena such as storms or hails.

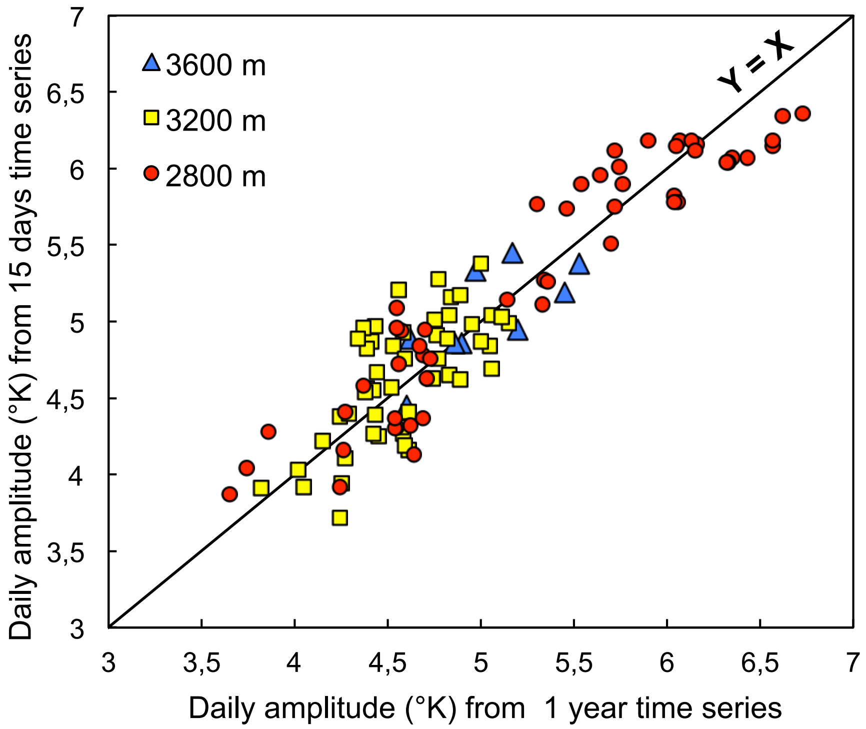

Figure S4: Scatter plot of Fourier transform amplitude for the daily frequency of a 15 days air temperature time series vs. a 1-year air temperature time series at three elevations.
