# Supplementary material for: Strong Discrepancies between Local Temperature Mapping and Interpolated Climatic Grids in Tropical Mountainous Agricultural Landscapes
Source: PLoS One. 2014 Aug 20;9(8):e105541. doi: 10.1371/journal.pone.0105541 (PMC4139370; doi:10.1371/journal.pone.0105541)
Supplement: Appendix S5 — Fourier analysis description. (PDF) [file pone.0105541.s005.pdf]

Appendix S5: Fourier analysis description.

The Discrete Fourier Transform (DFT) used in this study was defined as follow:

$$X_k = \frac{1}{N} \sum_{n=0}^{N-1} x_n e^{-i2\pi k \frac{n}{N}}, \ \mathrm{k} = 0, \ldots, \mathrm{N}-1 \qquad \text{(eqn 2)}$$

where X_k, the Fourier transform complex coefficient, is the frequency domain representation of the signal time series x_n at the k_{th} frequency, N is the total number of samples of the time series, and i is the imaginary unit (see [44] for details). The amplitude A_d and the phase ϕ_d of the DFT are defined by definition (≜) as follows:

$$\mathrm{A_d} \triangleq |\mathrm{X_{k_d}}| \qquad \text{(eqn 3)}$$

$$\phi_\mathrm{d} \triangleq \arg(\mathrm{X_{k_d}}) \qquad \text{(eqn 4)}$$

The thermal amplitude allowed us to measure the thermal buffer effect in Kelvin (see Fig. 1) between air and canopy layers (β^p) and air and soil layers (β^s), by calculating the difference of the DFT amplitudes as follows:

$$\begin{cases} \beta^\mathrm{p} \triangleq \mathrm{A_d^a} - \mathrm{A_d^p} \\ \beta^\mathrm{s} \triangleq \mathrm{A_d^a} - \mathrm{A_d^s} \end{cases} \qquad \text{(eqn 5)}$$

where A_d^a, A_d^p and A_d^s are the DFT amplitudes at the daily frequency for air, air canopy and soil time series, respectively (see equation 3).

As we were interested in amplitude differences between air vs. air canopy and air vs. soil for maximum and minimum daily temperatures, we then defined the daytime (M) and night-time (m) temperature excursions between air vs. plant canopy ($\varepsilon^\mathrm{p}_\mathrm{M,m}$) and air vs. soil ($\varepsilon^\mathrm{s}_\mathrm{M,m}$) as follows:

$$\begin{cases} \varepsilon^\mathrm{p}_\mathrm{M} \triangleq \mathrm{A_0^a} - \mathrm{A_0^p} + \beta^\mathrm{p} \\ \varepsilon^\mathrm{s}_\mathrm{M} \triangleq \mathrm{A_0^a} - \mathrm{A_0^s} + \beta^\mathrm{s} \end{cases} \qquad \text{(eqn 6)}$$

$$\begin{cases} \varepsilon^\mathrm{p}_\mathrm{m} \triangleq \mathrm{A_0^a} - \mathrm{A_0^p} - \beta^\mathrm{p} \\ \varepsilon^\mathrm{s}_\mathrm{m} \triangleq \mathrm{A_0^a} - \mathrm{A_0^s} - \beta^\mathrm{s} \end{cases} \qquad \text{(eqn 7)}$$

with A_0 is the mean DFT value of the time series.

The phase allowed us to measure the thermal time lag τ_d expressed in minute in canopy (τ_d^p) and soil layers (τ_d^s) with respect to the air layer (see Fig. 1) by calculating the difference of the DFT phases as follows:

$$\begin{cases} \tau_d^p \triangleq \frac{24}{2\pi}(\phi_d^p - \phi_d^a) \\ \tau_d^s \triangleq \frac{24}{2\pi}(\phi_d^s - \phi_d^a) \end{cases} \qquad \text{(eqn 8)}$$

where ϕ_d^a, $\phi_d^p$and ϕ_d^p are the DFT phases at the daily frequency for air, air canopy and soil time series, respectively (see equation 4).

All Fourier analyses were performed in MATLAB R2011a (The Mathworks Inc., Natick, USA).
