# Supplementary material for: Strong Discrepancies between Local Temperature Mapping and Interpolated Climatic Grids in Tropical Mountainous Agricultural Landscapes
Source: PLoS One. 2014 Aug 20;9(8):e105541. doi: 10.1371/journal.pone.0105541 (PMC4139370; doi:10.1371/journal.pone.0105541)
Supplement: Appendix S6 — Seanonality measured on four year air temperature time series with Discrete Fourier Transform. (PDF) [file pone.0105541.s006.pdf]

Appendix S6: Seasonality measured on four-year air temperature time series with Discrete Fourier Transform.

	Mean amplitude of the discrete Fourier transform (°K)		
Period (days)	2800 m	3200 m	3600 m
91	0.14 (+/- 0.3)	0.15 (+/- 0.1)	0.12 (+/- 0.2)
182	0.41 (+/- 0.18)	0.49 (+/- 0.15)	0.43 (+/- 0.13)
364	0.94 (+/- 0.15)	1.01 (+/- 0.17)	0.96 (+/- 0.11)

Table S6: Mean amplitudes in Kelvin of the discrete Fourier transform at the seasonally frequencies (91, 182 and 364 days) of four year monitoring of air temperatures (recorded at 1 meter high with half an hour time step with the same shelter process describe above between 2008-2012) for the three altitudinal belts of the study area (2 replicates for each elevation).
