# Supplementary material for: Strong Discrepancies between Local Temperature Mapping and Interpolated Climatic Grids in Tropical Mountainous Agricultural Landscapes
Source: PLoS One. 2014 Aug 20;9(8):e105541. doi: 10.1371/journal.pone.0105541 (PMC4139370; doi:10.1371/journal.pone.0105541)
Supplement: Appendix S7 — Crop habitat composition survey used in the study area. (PDF) [file pone.0105541.s007.pdf]

Appendix S7: Crop habitat composition survey in the study area.

Crop habitat composition in the study area was measured in 85 independent locations at different altitudes between 2008 and 2012. The relative area of each crop type (in %) was visually estimated by two observers in a 100-m radius circle around each location. The mean of the two observations was then calculated. The phenological stage of each crop was also recorded.

Elevation	Potato	Broad bean	Corn	Alfalfa	Pasture
2800 m	20.9	13.4	18.4	16.5	30.8
3200 m	24.1	13.3	15.9	12.5	34.2
3600 m	27.3	9.1	6.6	7.6	49.4

Table S7: Mean crop composition (%) at three altitudes used to parameterize thermal landscape mapping (see Fig. 2 & 3 in the main document). The number of independent location were N = 43, 25, 17 at 2800, 3200, and 3600 m, respectively.
