# Supplementary material for: Strong Discrepancies between Local Temperature Mapping and Interpolated Climatic Grids in Tropical Mountainous Agricultural Landscapes
Source: PLoS One. 2014 Aug 20;9(8):e105541. doi: 10.1371/journal.pone.0105541 (PMC4139370; doi:10.1371/journal.pone.0105541)
Supplement: Appendix S8 — Local and global air mean temperature discrepancies mapping. (PDF) [file pone.0105541.s008.pdf]

**Appendix S8:** Local and global air mean temperature discrepancies mapping.

Differences in mean temperatures between local air temperatures (extracted from the Fourier transform) and the global coarse grain interpolated air temperatures from the WorldClim (*Δ* Air $_L$ - Air $_{WC}$) were mapped for the three studied grid cells (Fig. S8). Generally, local air temperatures were 1.4°K warmer than the global interpolated ones. Mean temperature discrepancies were of 0.3 ± 1°K, 1.7 ± 1.6°K and 2.3 ± 1.5°K at 2800 m, 3200 m and 3600 m respectively. For the three studied grid cells minimum of average temperature discrepancies was -2°K and maximum reached +8°K. As a consequence, 44.6 ± 3.4 % of the studied areas were either under-estimated or over-estimated by the global climatic models (+/- 1°K).

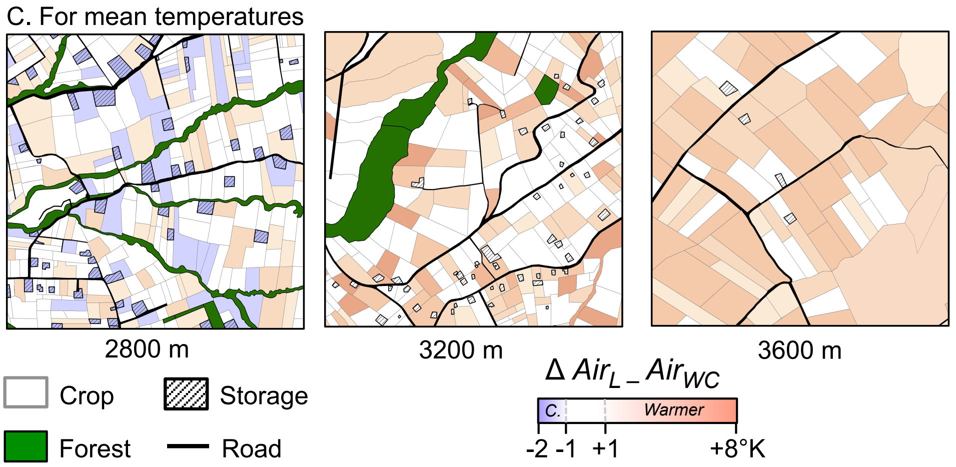


**Figure S8:** Maps showing the differences between the measured local air temperatures and the WorldClim interpolated temperatures for mean values *(Δ Air Local – Air WorldClim = Δ Air $_L$ – Air $_{WC}$)*. Blue colours indicate *Δ Air $_L$ – Air $_{WC}$* < 0, i.e area where local air temperatures are cooler than the ones gave by WorldClim, red colours indicate *Δ Air $_L$ – Air $_{WC}$* > 0, i.e. area where air local temperatures are warmer than the ones gave by the WorldClim, and white colours *Δ Air $_L$ – Air $_{WC}$* = 0 indicate areas where air WorldClim temperatures equate air local temperatures (± 1°C). The extent and position of each square is equal to the spatial resolution of the WorldClim database: 30-arc sec that is the equivalent of 0.86 km$^2$ for the study area. Each side of square has a 925 m length. The temperature dataset for storages was obtained from [26].
